# Medical Defence

**Published:** 1938

**Authors:** Clifford A. Moore

**Affiliations:** Surgeon to the Bristol General Hospital


### The Bristol Medico-Chirurgical Journal

"*Scire est nescire, nisi id me*
*Scire alius sciret.*"

SPRING, 1938.

### MEDICAL DEFENCE.

The Presidential Address, delivered on 13th October, 1937, at the opening of the Sixty-fifth Session of the Bristol Medico-Chirurgical Society.

BY

Clifford A. Moore, M.S., Ch.M., F.R.C.S.
*Surgeon to the Bristol General Hospital.*

The subject I have selected for my address is one which concerns us all, for with it may be closely bound up our future prosperity and happiness, our professional reputation, even our professional existence. I propose to draw on my experiences and give you some impressions gained from a good many years' service now, on the Council of the Medical Defence Union.

Most people know, no doubt, that that is not the only society which is concerned with such matters, for there is another, the London and Counties Medical Protection Society. If the Medical Defence Union can justly claim to be the older, the larger and the richer, no member of the other society need have the least fear that his affairs are not in safe hands, both from the point of view of skilled management and

B

absolute financial stability. I have no wish that what I have to say should be in any sense propaganda on behalf of my own society, but that it should be taken as typical of Medical Defence in general. My experiences could be, no doubt, exactly duplicated by anyone familiar with the inner working of the other society.

It is not without interest to look back and inquire how it comes about that there should be two societies with the same objects, and doing precisely the same kind of work. To understand this, we must go back to the year 1892, which was seven years after the foundation of the original society. In that year a member of the Council differed from his colleagues over the conduct of the society's affairs, he found himself in a minority of one, and he left the society to found a rival organization, the London and Counties Medical Protection Society. Since that time, several efforts have been made to bring about an amalgamation of the two organizations, but without result. Opinions probably differ as to whether amalgamation is desirable, or whether efficiency is not rather promoted by having two societies in healthy rivalry. The financial position of each is so very strong that it is doubtful if anything would be gained by uniting them into one.

I have called the two societies rivals, but I should hasten to add that they are friendly ones and work in the closest co-operation. When action is threatened or commenced against a member of the Profession who is in partnership, it is practically always brought against the firm, not against the individual, even if one person only was concerned in the matter complained of. It naturally happens quite often in such cases that one member of the firm belongs to one society and another to the other. Such cases cause no difficulty at all, for they are dealt with by a standing joint committee composed of an equal number from each society. The Committee works according to set Rules of Procedure, and no serious difficulty has, so far as I am aware, ever arisen.

An inquiry as to how the necessity arose for some form of organized medical defence takes us back more than half a century, but although the original society was actually founded in the year 1885, the events which gave rise to it occurred two years earlier. In the year 1883, two practitioners of Dulwich—Doctors Bower and Keates—were sued for negligence and prosecuted for manslaughter in connection with their treatment of a child suffering from diphtheria. The child died, and it was alleged that it was owing to the instructions given to the father that he himself contracted the disease. Both charges failed, and at the end of the hearing the Court expressed the opinion that the case represented "persecution rather than prosecution." In spite of their victory, the defence cost the two doctors more than one thousand pounds. Widespread sympathy with them was expressed throughout the profession, and a meeting was called, presided over by Sir William Jenner, to see what could be done to help them. The result was that the amount required to pay for the defence was subscribed at once.

This case brought the question of medical defence very much to the fore, and many resolutions were sent in to headquarters by branches and divisions of the British Medical Association appealing for the provision by the Association of some form of cooperative medical defence. Counsel's opinion was, however, that the funds of the Association could not be properly used in this way. After the reconstruction of the Association in 1903 the question was again raised, but it was again decided not to interfere with the work of the societies by competing with them. Most members will probably think that this decision was a wise one.

The actual prime movers in the matter were, curiously enough, not medical men at all, for it was a body of seven laymen who, on the 23rd October, 1885, registered the Medical Defence Union as a company limited by guarantee, under the Companies Act of 1862. Meetings were held in numerous provincial centres, and it was in Birmingham that most enthusiasm was shown; and it was from a meeting held there that the Union, as we know it, actually sprang. The first President was Lawson Tait, the famous gynæcologist. Later in the same year the infant Union was all but wrecked by the misappropriation of its funds by an official, but the delinquent was brought to justice and the Union weathered the storm. With the driving power still largely supplied from Birmingham, the Union began to develop on the lines of to-day. It first appeared in the rôle of Plaintiff in 1889, when it successfully sued the Great Western Railway Provident Society for the wrongful dismissal of one of its surgeons.

It will probably be thought that the time has now come for me to give up delving into the past and to deal with some practical problems of medical defence as it affects us to-day. While listening to the discussions of these problems month after month, three things keep recurring to one's mind. Firstly, how any doctor engaged in any kind of practice can be so utterly foolish as to neglect to insure himself against charges of negligence, which is only one aspect, albeit the most important one, of the subject. A charge of so-called negligence may arise out of the blue at any moment. However frivolous and without foundation in fact, it needs much trouble, and possibly much money, to deal successfully with it. It is to be noted that many of the more frivolous claims are merely a dodge to attempt to evade payment of an account. Your Society does not claim to be in any sense a debt-collecting agency. If your patient merely refuses to pay his account, or takes no notice when you send it in, the County Court is the place for him. Only let him, however, make a complaint about your treatment as an excuse for refusing or neglecting to pay, and his challenge will be quickly taken up.

The other two points to which I referred are how foolishly some doctors can act, and how ungrateful, grasping and unscrupulous can be a minority of patients. Let me hasten to emphasize that it is only a very small minority who act in this way, for it is in only a comparatively few cases that anything occurs to disturb seriously the atmosphere of confidence and trust which should exist between doctor and patient.

As I have already mentioned, a great part of the work of the defence societies deals with charges of negligence, justified or otherwise, trivial or grave. It must be remembered, however, that this forms only a small fraction of their activities. Only a small proportion of the cases dealt with ever reaches the Courts or gets into the papers. Nevertheless, the amount of trouble, anxiety and expense saved to their members in the aggregate is enormous.

To take the general practitioner first, as the most numerous and most important section of the profession, it may be interesting to set out some of the ways in which trouble can overtake him. He is not concerned as a rule in the big cases of malpraxis which get into the papers, but he can, and he does get into frequent difficulty in his work under various Acts of Parliament, for instance, the Public Health, Midwives, National Insurance and Lunacy Acts, in disputes with various Public Authorities, and in numerous other ways.

Hypodermic needles cause any amount of trouble. The needle breaks off and remains in the patient's tissues and very frequently trouble follows. So often does this happen that one society has issued a set of model rules, which includes the advice never to use a hypodermic needle a second time. This is, indeed, a counsel of perfection, and a more economical and almost equally reliable rule is never to insert the needle up to the hilt. It is always at the rusted joint where the needle is inserted into the mount that the break occurs. Enough should always be left projecting to enable it to be retrieved should a break occur. Intramuscular needles, in particular, should always be of ample length to allow this to be done.

The following is an interesting case bearing upon this point. A doctor was giving an intramuscular injection to a somewhat nervous patient when she made a sudden movement, and the needle broke off and disappeared. The doctor said nothing and allowed the patient to travel home by bus, a distance of two miles. A few days later he was again called in because the patient complained of some tenderness at the site of injection. On this occasion he told the patient's husband what had occurred and arranged for the removal of the needle at the local hospital, which was successfully done.

A charge of negligence was made and an action for damages started. This was heard in the County Court by a judge, sitting without a jury. He awarded five guineas damages on the ground that the patient had not been informed of the mishap and had been permitted to travel home by bus. On the main issue—that is, of negligence in the performance of the injection—judgment was given for the defendant. This finding appears to mean that the breaking of a hypodermic needle and its loss in the tissues is not negligence, but that to fail to inform the patient of the fact is.

A word of warning must be given here on the question of X-rays. There are still far too many cases of suspected fracture—or unsuspected, for that matter—where an X-ray examination has been omitted and where it has been subsequently demonstrated that a fracture is present. It is highly dangerous to treat a contused hip without an X-ray ; in a large proportion a fracture of the neck of the femur has occurred. If you have a case in which you think an X-ray is advisable and the patient refuses, on the ground of expense, or the effort required, or for any other reason, make a careful note in writing at the time in case there is trouble later. It is becoming almost *prima facie* evidence of negligence to have omitted an X-ray in a case where a fracture eventually turns out to be present.

The consulting physician seems to get off very lightly, for I can recollect scarcely a case in which one was seriously involved. The Mental Treatment Act of 1930 must have removed a lot of anxiety from the minds of those whose duty it is to certify persons of unsound mind. This Act provides that no action can be brought against any person who has acted in pursuance of the Lunacy Acts unless the leave of the Court is first obtained, and such leave shall not be given unless the Court is satisfied that there are substantial grounds for the contention that the person against whom it is sought to bring the proceedings has acted in bad faith or without reasonable care.

I should perhaps mention here what is frequently a physician's operation, namely, the injection of alcohol into the Gasserian ganglion in the treatment of trigeminal neuralgia. The results of the alcohol spreading beyond the ganglion and affecting other cranial nerves are disastrous indeed, and there are many cases where it has done so. In my opinion this manœuvre should be left to those, be they physician or surgeon, who get sufficiently frequent opportunities of "keeping their hands in." The dangers of a general anæsthetic do not seem to be sufficiently recognized: for it will mask the area of anæsthesia following a preliminary injection of novocain into the ganglion which is such a valuable safeguard against a catastrophe of this nature.

Those engaged in the academic teaching of anatomy, physiology or pathology would seem to be reasonably safe, but if I were so engaged I think I should give myself the benefit of the doubt and insure. The clinical pathologist can certainly get into trouble, and so can the dermatologist, for radium and high-voltage X-rays are getting more and more important as means of treatment, and it sometimes happens that mishaps occur.

In the ear, nose and throat department, cases occur in which a lot of harm has been done in the naso-pharynx when sloughing has followed a tonsil or adenoid operation. Other cases concern the facial paralyses after operations on the mastoid, while disastrous damage to the optic nerve has followed on an operation on the nasal sinuses.

Here perhaps is an appropriate place to mention the explosions which have occurred in operating theatres when a mixture of oxygen and ether has been used as the anæsthetic. This mixture is so highly explosive that a minute spark from a two-volt bulb worked from a dry battery has been sufficient to ignite it, and several lives have been lost in this way. A mixture of ether and air is much less highly explosive, and it does not seem to be appreciably less efficient.

And now I come to what is to some of us the most important aspect of the question, the general surgeon and his liability to charges of malpraxis or negligence. In this connection the swab looms large and I am sometimes tempted to wonder if it is a common thing for a surgeon to open the abdomen and *not* to leave a swab or an instrument inside. In comparison, of course, the number of such disasters is very small, but in the aggregate it is large.

Some of these swabs are not left in the abdomen at all, but somewhere else. In one case a tampon was left in the vagina after a curettage, where it was over-looked, to be discovered a week or so later by the nurse. Actually, of course, it had done no harm, but it led to a charge of negligence. Quite large swabs and packs have been left in the axilla after breast operations, and some are left in the gastro-intestinal tract itself. In a recent case a surgeon did an extensive and successful partial gastrectomy and the patient left the Hospital. Shortly afterwards he began to vomit, and one day in an attack of vomiting he brought up a foreign body. This proved to be a gauze pack, which when wet and compressed was as large as an egg, but when dried, fluffed out and put on the stretch, it was a strip of gauze twelve feet long and six inches wide. Imagine an indignant counsel for the plaintiff holding this up at arm's length before the eyes of a sympathetic jury, and demanding compensation almost literally by the yard. Actually the patient had suffered only temporary inconvenience, and a small *ex gratia* payment disposed of his claim.

I must ask those who do not do abdominal operations to bear with me while I refer in greater detail to this swab question. It is often argued, and most people would think justly, that the surgeon forms only one of a team: that he cannot possibly be personally responsible for every detail of an operation, and that the counting of swabs and instruments must be the responsibility of others. This sounds reasonable, but unfortunately it has never been established as a principle of English law. To deal with this point I must refer to one or two decided cases, and from them it would appear that overseas surgeons are more fortunate than their colleagues at home in this respect.

The best known case is that of Van Wyk *v.* Lewis, decided in 1924 in the Supreme Court of South Africa, where the Court found that the surgeon was not responsible for a swab left in the abdomen at a laparotomy. The Judge's words were: "The surgeon does not ensure that he will be responsible for the misfeasance of the nurse: to make him so would be to make his position intolerable."

In the case of Ingram *v.* Fitzgerald, heard last year in the New Zealand Court of Appeal, the facts were somewhat different but the principle was the same. It was not a swab case, but one in which a theatre nurse, intending to paint a patient's abdomen with tincture of iodine, used instead iodized phenol which had been used at an earlier stage of the same operation. The mistake was discovered at once and prompt steps were taken to rectify it. It appears that no very serious damage was done, but an action for damages followed nevertheless. The Court found that in the particular circumstances the surgeon was not liable for the negligence proved against the nurse, and judgment was given for the defendant.

The best-known case in this country is that of Crotch *v.* Miles, which was tried in 1930. Here a patient sued a surgeon for negligence, alleging that he had left a pair of artery forceps in her abdomen. Lord Hewart, the Lord Chief Justice, put it to the jury that they must consider whether the surgeon ought to have known or found out for himself if anything was missing, or whether he was entitled to rely on the theatre-sister. He also expressed the opinion that it was unreasonable to expect the surgeon to count anything in the course of the operation, but that after the wound was stitched up it was reasonable to expect him personally to satisfy himself that nothing is missing. In point of fact, this case decided nothing, for the plaintiff had had a previous laparotomy in France and the jury was not satisfied when, in fact, the forceps were left in the abdomen. Judgment for the defendant followed.

The present position may be summed up in the words of the *British Medical Journal* as follows:—"The test must apparently always be, in the particular circumstances of the case, could the surgeon reasonably have been expected to supervise or check the actions of the assistant, and to intervene if a mistake was committed?" It is quite certain that in every case the defence would have to be prepared with evidence that a definite system is in force in the Hospital concerned for checking instruments and swabs, which in ordinary circumstances should suffice to prevent these accidents. Human fallibility will always be with us, and it is impossible to foresee a time when these misadventures are entirely things of the past.

There is one point which arises when the defence of such cases is under consideration, and that is the fact that to defend the surgeon it is almost inevitable in many cases that an attempt is made to transfer the blame to the shoulders of the nursing staff or some other subordinate, which appears unchivalrous. These cases, however, when they reach the Courts must be dealt with according to the Law, and not by any other consideration. Some will think that the modern highly-trained nurse ought to be able to stand on her own feet and shoulder every responsibility which her status puts upon her. Any damages awarded would rarely be against the nurse personally, but against the Hospital where she was employed. Many Hospitals are, and all should be, insured against accidents of this kind. It is time I left this subject now and passed on.

Up till the middle of last century the dependants of a person who had died as a result of the wrongful act of another had no right of action at all in respect of the death of the deceased. In the year 1846, however, the Fatal Accidents Act—better known as Lord Campbell's Act—appeared on the Statute Book. This Act created a limited right of action in that the dependants could claim in point of damages the actual monetary loss sustained by such a death, although they could not claim what might be called sentimental damages. For instance, a parent could only claim in respect of the death of a child if it could be proved that the parent had expectations from the child, either in contributions to the household expenses or otherwise. In any event, the amount that could be claimed was quite small, being limited to £250.

With this small exception, the old principle of British Law has held—*Actio personalis moritur cum persona*—which means that the right of personal action dies with the individual, whether plaintiff or defendant. To make a concrete case of it, the legal representatives of a deceased patient could not take into the Courts a grievance which they might think they had against the medical attendant of the deceased. Similarly, an aggrieved patient could not pursue a claim against the estate of his deceased medical attendant.

This position has been completely altered by the Law Reform Act of 1934, which extended the provisions of the earlier Act so as virtually to put the dependants in the shoes of the deceased so far as the right to claim damages was concerned. The aggrieved relatives of a deceased patient *can* now sue his medical attendant for negligence: moreover, an aggrieved patient can now sue the legal representatives of his deceased medical attendant. Members may have seen reported in the papers a recent case in which this principle doubly applied, for the relatives of a deceased patient obtained heavy damages for negligence against the estate of his deceased medical attendant. This fundamental change in the legal position has been quickly recognized by the defence organizations. They are prepared to assume responsibility for defending the interests of the dependants of a deceased member in case trouble of this sort should arise. I need hardly say that it is essential that the deceased member should have been in benefit both at the time the incident occurred from which the charge arose, and also at the time of his death.

I now turn to a branch of the work which fortunately affects few of us. Nevertheless, to that few it is of vital importance. This is the handling of cases which come before the General Medical Council. In the average case of this sort it is essential that the interests of the doctor whose conduct is in question should be represented by a solicitor, and I cannot help thinking that the solicitors to the defence societies carry more weight than others, however eminent or experienced. At any rate, it cannot be denied that these same solicitors are very useful to the Council itself in undertaking the prosecution of those charged with carrying on unqualified practice. It must be remembered that these prosecutions are never undertaken by the Council itself, but are handed over to the defence societies.

It is of interest to see what happens to the unfortunates who are hailed before the C.G.M. In one year, chosen at random, there were eleven members of one society who found themselves in the unfortunate position of being charged with various offences before the Council. Of these eleven, in three cases the written explanation was accepted and no further action was taken. In five more the defence was successful—in three because the charge was held not to be proved, and in two more the offence was held to be proved but erasure from the Register did not follow. The remaining three were struck off the Register.

I must not omit mention of the Coroner's Court, and the various ways in which help may be required there. To give an example, requests are often received for representation at an inquest by a doctor who fears that his conduct of the case may be called into question. Such cases have to be very carefully considered, for the mere fact, if known, that a doctor is legally represented at an inquest might suggest at once to some people that he must have a guilty conscience. If it is reasonably certain that the conduct of a doctor will be severely criticized, legal representation will be given as a matter of course. In other cases it is generally unwise, because if it appears desirable in the course of the proceedings, an appeal to the Coroner to adjourn the inquest is bound to be listened to. In other cases a solicitor can be present in case his services should be required, his presence and the purpose of it having been previously and privately notified to the Coroner.

We in Bristol are fortunate in that for many years the relationship between the City Coroner and the local profession has been of the happiest, as it must be if the Coroner's Court is to function efficiently. Other districts, however, are not so fortunate, and trouble with the Coroner himself is not unknown. In some Coroner's Courts the proceedings are liable to be conducted in a highly irregular way, and the Coroner has a distorted idea of his powers. The following case illustrates the sort of thing that may happen. A Coroner requested the doctor concerned to make a post mortem before the inquest. In the course of the inquiry he said it was clear from the evidence that there had been no real need for a post mortem, for the doctor could quite well have certified the cause of death without one, and he eventually refused to pay the fee. It was only after considerable pressure that he climbed down and paid up. I might mention that in cases like this, where the Coroner is obviously exceeding his powers, an appeal to the Coroners' Society generally has a good effect.

Coroners are, of course, nearly always solicitors, and we sometimes have trouble with other members of that profession. A doctor is frequently asked to supply a written report on a patient who has been injured, for instance in a street accident. The request for the report contains some such phrase as "Your fee will be paid," or it merely asks for the amount of the fee to be stated. The plaintiff, often a person of no means, loses the case, and in due course you ask for your fee: you are told by the solicitor that he has no funds out of which to pay the fee, and you are referred to the plaintiff. Of course, you get nothing.

In any case of doubt you will be wise to decline to part with any information or report until you get either the fee in advance or the solicitor's personal guarantee to pay it: in that case, if he fails to pay, he can be dealt with. The defence societies, together with the British Medical Association, have recently taken up this question with the Incorporated Law Society, and they received a very sympathetic hearing. There is no doubt that any solicitor who has given a personal guarantee to pay and fails to do so can be speedily brought to book.

Before I turn to some remarks about medical defence in general, there is just one more aspect of the defence societies' work which requires a few words, and that is the suppression of unqualified practice. As I have said, that is never undertaken by the General Medical Council, the societies themselves undertaking the prosecution of offenders. It has taken a long time and several prosecutions to establish the fact that it is illegal for anyone but a registered medical practitioner to describe himself, or herself, as a physician or surgeon, whether the word is preceded by a qualifying adjective or no. A plate describing the unqualified owner as a "manipulative surgeon" or an "osteopathic physician" is not allowed. It has happened more than once that one of these gentry has been prosecuted unsuccessfully, and it is amusing to note the effect on his colleagues. Plates which had been taken down under threat of prosecution go up again, and their owners, hitherto submissive, once more become defiant. The question is pretty well settled now, and even if a bench of magistrates does dismiss a case of this sort, it is certain that a Higher Court will override the decision. Possibly some member of this Society knows of a plate somewhere which bears the words "physician" or "surgeon" with a qualifying adjective. If he will be so kind as to furnish me with particulars, I shall have much pleasure in setting the machinery in motion which will cause its disappearance.

I think I have dealt with most of the chief points which arise in this connection, and I will only add a few remarks in general on the obligations which lie on members of the profession who would feel themselves reasonably safe from serious risk. What must they do to feel safe? The principal thing, having joined a defence society, is to take every care to keep yourself in benefit—or, in plain language, avoid getting into arrears with your subscription. This is doubly important, for the rules provide that to be eligible for assistance you must be in benefit both when the incident occurred from which the claim arose, as well as when the claim was actually received. These two dates may be separated by a period of years. I should make it clear that your subscription is due on each first of January, but you are allowed a month's grace, so that you are continuously in benefit if you pay not later than 31st January. Delay your payment, on the other hand, only until 1st February, and you are out of benefit during the whole month of January, and you may be refused assistance over any claim which originated during that month. I emphasize the word *may*: until quite recently it would have been *must.*

It must always be remembered that these societies are Limited Companies, and their funds have to be administered strictly in accordance with the Articles of Association. The funds are provided by members for the benefit of members who are in benefit. They cannot be disbursed on compassionate grounds, nor to meet cases, however hard, which do not come within the rules. Until quite recently help must be refused to those out of benefit, but the Articles of Association have been recently modified by the insertion of the words "except under special circumstances." One can imagine a special circumstance as being held to have arisen in the case of a man who had let his subscription lapse for a few weeks, or a month or two, and who had a claim made upon him during an out-of-benefit period. It would very likely be looked upon as a special circumstance if an examination of the records showed that he was in the habit of paying up promptly, and was not a regular defaulter, as so many unfortunately are.

If you want to feel quite safe, you should join a society as soon as you are qualified. It is cheaper for one thing, for an entrance fee is waived in the case of the newly qualified. If you are not a member during your period of hospital residence, your choice of posts is limited, for many hospitals wisely insist on their residents being protected. The ways in which hospital residents can get into trouble are many, but I will only mention one example. A large proportion of intravenous injections of various drugs are given by residents. This applies to some of the various drugs which are used for intravenous urography, which are gaining an unenviable notoriety for causing trouble. The serious ulceration which sometimes follows faulty technique is, of course, well known, but cases of thrombosis in the injected veins and of trouble with the median nerve are not so well known.

Another sound rule is not to be in too much of a hurry to resign your membership immediately on retirement from practice. Remember that an incident may come to light which occurred months or years before your retirement. If the claim is only made after you have given up your membership, you are out of benefit, and in the cart. A special provision is made for those who have finally retired by giving them lifelong protection for a lump-sum payment. This varies with the number of years of active membership, but the maximum is three guineas.

In this connection I feel bound to mention just one more Act of Parliament, the Statute of Limitations. There is more than one such Statute, but the one which applies to alleged acts of negligence occurring in medical practice dates back to the year 1623. This Act lays it down that no claim for damages for a wrong done can be made unless it is made within a period of six years from the date of the injury. If you leave a pair of artery forceps in a patient's abdomen and it does not come to light until more than six years have elapsed, the Statute of Limitations is a perfectly good defence. It would undoubtedly save you from being cast in heavy damages, but it would not prevent a claim being made against you: even if it were only bluff—and many claims are—it would mean a lot of worry and expense to deal with it. The lesson to be learned from this is that you should maintain your membership for even longer than six years after your final retirement from practice. The plan I have mentioned above allows you to do this with a minimum of trouble and expense, and so to secure permanent peace of mind.

Every partner in a firm should be completely protected, for as I said above, claims for damages are nearly always made against a firm, not against the individual partner, even though one individual only is concerned in the incident complained of. Even if he is protected himself, his society will naturally not stand the whole racket if action is taken against the firm, if one or more members of it are unprotected. Let me once more emphasize that there is no need for both, or all, to be members of the same society. If all are protected there will be no difficulty in defending the case.

I have laid special stress on this question of keeping in benefit, for it is so important, but there is another point to be considered which is little less so. It is to take great care to give nothing away, and to make no admissions if you do have a claim made against you. Do not discuss the matter verbally, much less put anything in writing. If you get a letter of complaint about anything of the sort, do not answer it yourself, but send it straight to your society for advice as to how it should be dealt with. The defence of many a case has been hopelessly compromised at the outset by unwise handling, and by only making application to the society when things become serious. Expert handling in the early stages is of vast importance, and it may make all the difference between quashing a threatened claim and its development into a serious affair. Never hesitate to apply to your society at the slightest suggestion of trouble. Remember that if you compromise the defence of a case in the way I have mentioned you may be refused help altogether later, or asked to share the expenses, which may be very large.

I should mention that the degree of culpability varies a good deal, from the mildest expression of regret—natural but unwise—that harm has come from some action of yours, to the ingenuous individual who gives the whole case away in a few well-chosen admissions. He says how sorry he is that his treatment has been followed by such deplorable consequences, and how much he sympathizes with the patient. He adds that fortunately he is insured against such mishaps, and that generous compensation for the harm done is available and will be forthcoming. He forgets one thing: that the promised compensation is to be made, not out of his own pocket, but out of funds which are administered under strict rules, one of the most important of which he has broken. He is likely to find that his optimism is ill-timed and his generosity misplaced.

I have by no means exhausted my subject, but I think I have touched on the most important items of a subject in which I take a great interest. I need only add that if any member of this Society ever finds himself threatened with trouble of the kind I have been discussing, even if it is only to the extent of the vaguest of threats—and many serious cases start with vague threats—I shall be only too pleased to put at his disposal such knowledge of the subject as I have acquired.

